# Pediatric gigantism due to ectopic growth hormone releasing hormone secretion from a metastatic bronchial neuroendocrine tumor

**DOI:** 10.1210/jcemcr/luag304

**Published:** 2026-11-06

**Authors:** Fernando Vidal-Ostos De Lara, David Sanchis-Pascual, Lorena Hernández-Rienda, María Isabel Del Olmo-García

**Affiliations:** Department of Endocrinology and Nutrition, Hospital Universitario Príncipe de Asturias, Madrid 28806, Spain; Department of Endocrinology and Nutrition, University and Polytechnic Hospital La Fe, Valencia 46026, Spain; Department of Endocrinology and Nutrition, University and Polytechnic Hospital La Fe, Valencia 46026, Spain; Department of Endocrinology and Nutrition, University and Polytechnic Hospital La Fe, Valencia 46026, Spain; Medicine Department, University of Valencia, Valencia 46010, Spain

**Keywords:** gigantism, ectopic growth hormone–releasing hormone secretion, bronchial neuroendocrine tumor, pituitary hyperplasia, somatostatin analogs, peptide receptor radionuclide therapy

## Abstract

Pediatric gigantism due to ectopic secretion of growth hormone–releasing hormone (GHRH) is exceptionally rare. Most pediatric reports have involved pancreatic neuroendocrine tumors in the context of multiple endocrine neoplasia 1 (MEN1). We describe a female patient diagnosed at 9 years of age with a typical bronchial neuroendocrine tumor and subsequently found to have diffuse hepatic and skeletal metastases, accelerated linear growth, and acromegalic features. Evaluation demonstrated markedly elevated plasma GHRH, growth hormone, and insulin-like growth factor 1, with diffuse pituitary hyperplasia on magnetic resonance imaging and no discrete pituitary adenoma. Genetic testing for MEN1 was negative. Management required repeated surgery, first-generation somatostatin analogs, dopamine agonist therapy, pegvisomant, pasireotide, and peptide receptor radionuclide therapy with lutetium-177 DOTA-Tyr3-octreotate. Pasireotide achieved improved biochemical control, while peptide receptor radionuclide therapy contributed to long-term structural stability. After 23 years of follow-up, the patient remains clinically well with stable tumor burden and controlled somatotropic axis. This case highlights the natural history of a pediatric-onset GHRH-secreting neuroendocrine tumor and highlights the efficacy of aggressive, multidisciplinary management in achieving long-term survival.

## Introduction

Gigantism and acromegaly are most commonly caused by growth hormone (GH)-secreting pituitary adenomas, or somatotropinomas [1]. Growth hormone excess increases hepatic insulin-like growth factor 1 (IGF-1) production, which mediates accelerated linear growth in children and many metabolic and skeletal manifestations in adults [2]. Ectopic secretion of GH-releasing hormone (GHRH) by neuroendocrine tumors is an exceptionally rare cause of GH excess, accounting for less than 1% of cases [3].

Ectopic GHRH secretion should be considered when pituitary imaging shows diffuse pituitary enlargement without a discrete adenoma [1, 3]. In adults, bronchial and pancreatic neuroendocrine tumors are the most frequently reported sources; pediatric presentations are rare and have most often involved pancreatic neuroendocrine tumors in the setting of multiple endocrine neoplasia 1 (MEN1) [4, 5]. We report a case of pediatric-onset gigantism caused by ectopic GHRH secretion from a metastatic bronchial neuroendocrine tumor and describe the long-term multimodal management over more than 2 decades of follow-up.

## Case presentation

The patient is a 32-year-old woman who was initially diagnosed at 9 years of age with a 4 × 4 × 3-cm solid pulmonary mass in the right lower lobe after presenting with cough and hemoptysis (Fig. 1A). She underwent right middle and lower lobectomy. Histopathologic examination confirmed a differentiated bronchial carcinoid tumor (typical neuroendocrine tumor), with a low mitotic index (<5 mitoses per 10 high-power fields) and no necrosis (Fig. 1B).

**Figure 1:**
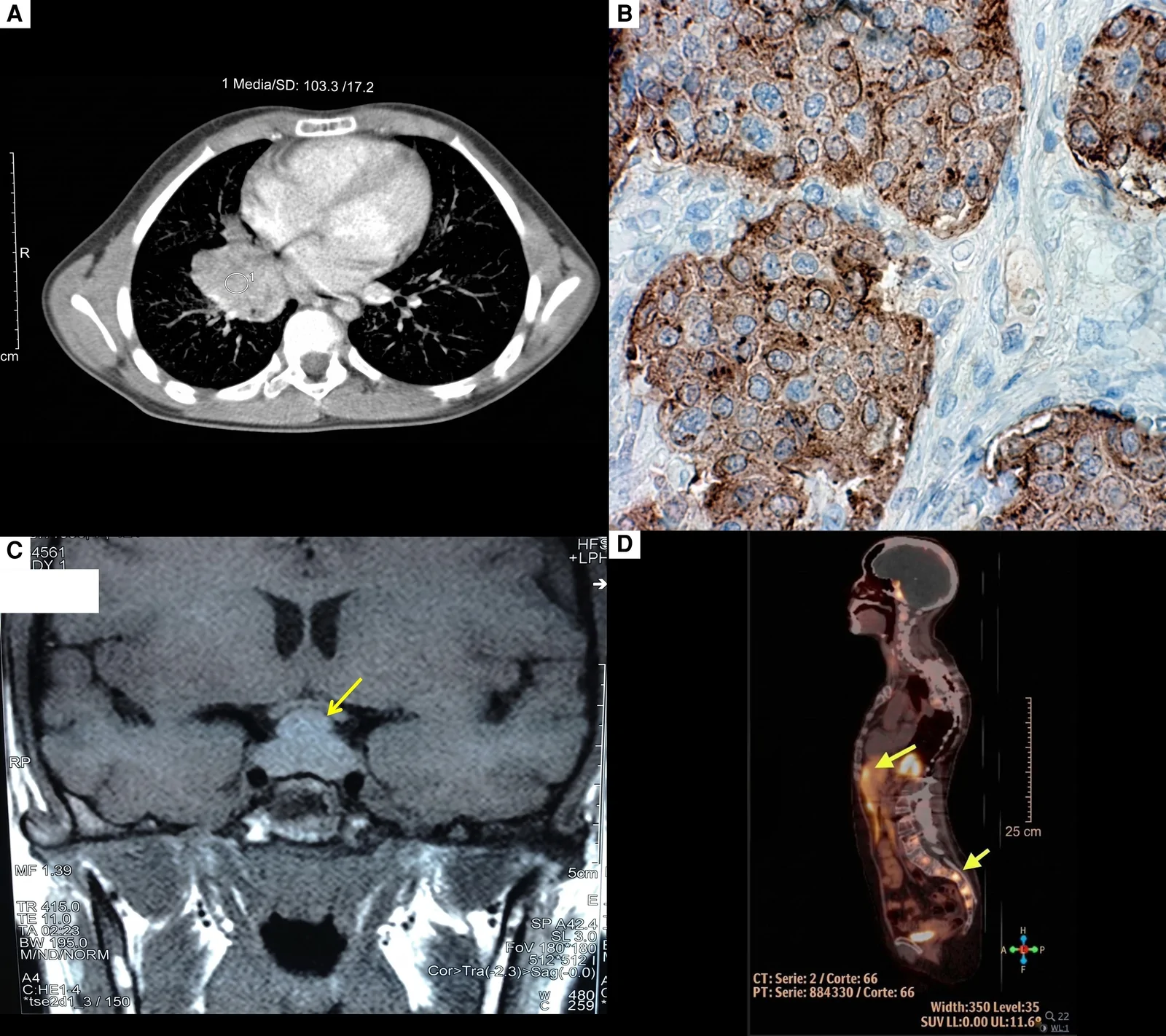
Diagnostic imaging and histopathologic findings. (A) Thoracic computed tomography at diagnosis showing a 4 × 4 × 3- cm solid mass in the right lower lobe. (B) Histopathologic examination (×40) of the bronchial tumor showing positive immunohistochemistry for chromogranin, enolase, and synaptophysin. (C) Brain magnetic resonance imaging performed at 12 years of age showing diffuse pituitary enlargement (1.7 × 1.0 × 1.2 cm) with suprasellar extension, lateral displacement of the cavernous carotid arteries, and compression of the optic chiasm; no discrete pituitary adenoma was identified. (D) Sagittal 68Ga-DOTATOC PET/CT showing multiple foci of avid radiotracer uptake in the axial skeleton, consistent with somatostatin receptor–positive bone metastases. Small arrows indicate representative abnormalities.

During oncologic follow-up, disease progression became evident, with hepatic and diffuse bone metastases involving the humeri and pelvis. At 11 years of age, she was referred to pediatric endocrinology because of tall stature (>97th percentile, +2.6 SD). The mid-parental target height was approximately 161 cm (paternal height ∼171 cm and maternal height ∼164 cm), well below her growth trajectory, which tracked at and above the 97th percentile and reached a final adult height of approximately 170 cm (Fig. 2).

**Figure 2:**
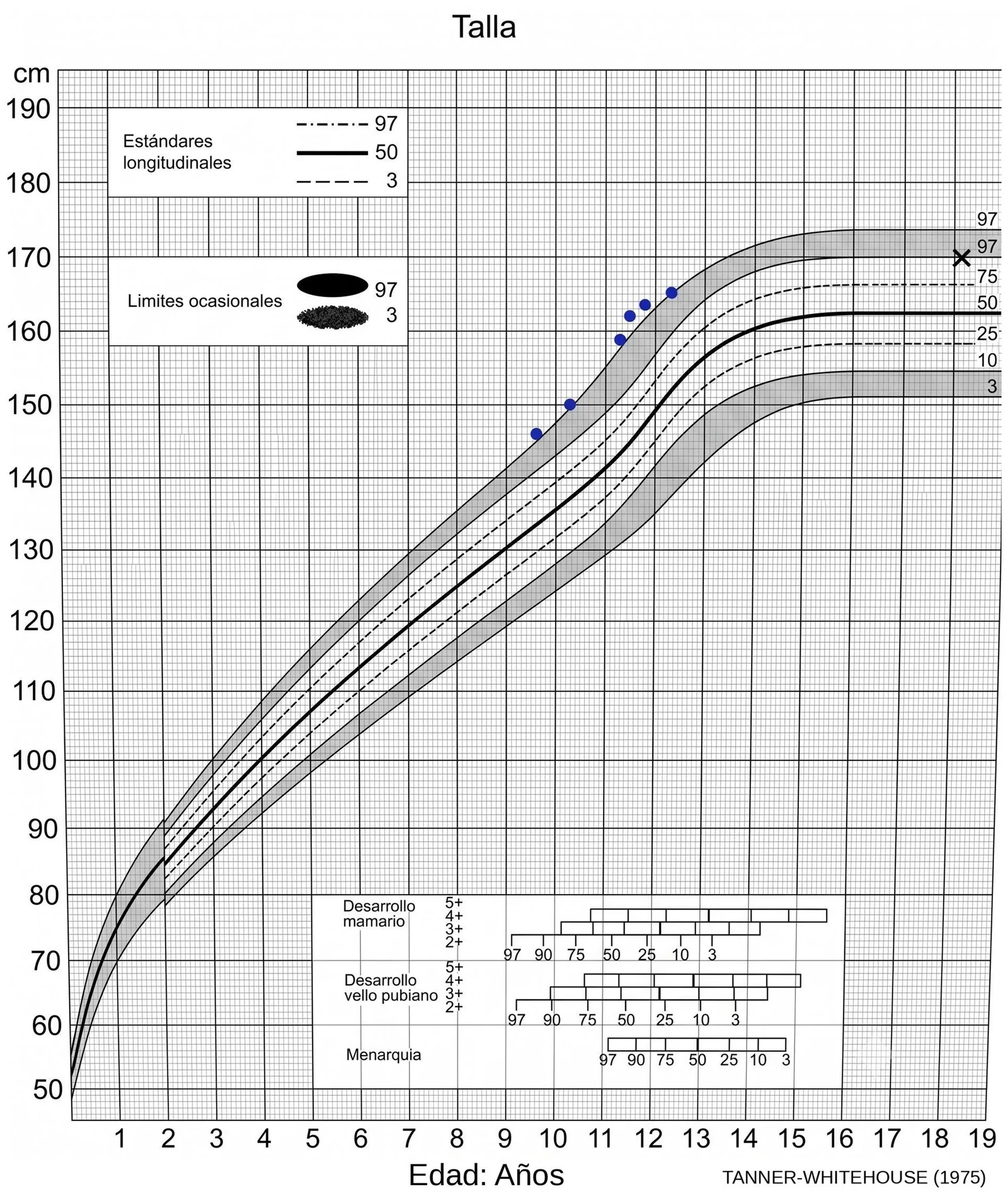
Growth chart showing progressive height acceleration during childhood, with stature exceeding the 97th percentile from approximately 10 years of age. The calculated mid-parental target height was approximately 161 cm (father: 171 cm; mother: 164 cm), substantially below the patient's final adult height. Earlier childhood growth records (before 7 years of age) were unavailable.

## Diagnostic assessment

Baseline hormonal evaluation confirmed severe hypersomatotropism. The serum GH concentration was 12.9 ng/mL (Système International [SI]: 12.9 µg/L) (reference range, 0.06-5.0 ng/mL [SI: 0.06-5.0 µg/L]), and the serum IGF-1 concentration was 1330 ng/mL (SI: 174 nmol/L) (reference range, 20-200 ng/mL [SI: 2.6-26 nmol/L]) Table 1. The serum prolactin concentration was also increased at 80.9 ng/mL (SI: 1715 mIU/L) (reference range, 4.0-15.2 ng/mL [SI: 85-322 mIU/L]) (Table 1).

**Table 1:** Key biochemical findings

Analyte/assessment	Result, conventional units	Result, SI units	Reference range, conventional units	Reference range, SI units
Growth hormone	12.9 ng/mL	12.9 µg/L	0.06 to 5.0 ng/mL	0.06 to 5.0 µg/L
Insulin-like growth factor 1	1330 ng/mL	174 nmol/L	20 to 200 ng/mL	2.6 to 26 nmol/L
Prolactin	80.9 ng/mL	1715 mIU/L	4.0 to 15.2 ng/mL	85 to 322 mIU/L
Follicle-stimulating hormone	0.8 mIU/mL	0.8 IU/L	1.5 to 12.4 mIU/mL	1.5 to 12.4 IU/L
Luteinizing hormone	<0.07 mIU/mL	<0.07 IU/L	1.7 to 8.6 mIU/mL	1.7 to 8.6 IU/L
Estradiol	<12 pg/mL	<44 pmol/L	5 to 36 pg/mL	18 to 132 pmol/L
Growth hormone–releasing hormone	962 ng/dL	9620 ng/L	<60 ng/dL	<600 ng/L
24-Hour urinary 5-hydroxyindoleacetic acid	9.2 mg/24 hour	48 µmol/24 hour	<12 mg/24 hour	<63 µmol/24 hour
Current insulin-like growth factor 1	163 ng/mL	21.3 nmol/L	93 to 311 ng/mL	12.2 to 40.7 nmol/L
Current chromogranin A	243.6 ng/mL	243.6 µg/L	<100 ng/mL	<100 µg/L

Abbreviation: SI, Système International.

Gonadotropin testing showed a prepubertal profile, with follicle-stimulating hormone 0.8 mIU/mL (SI: 0.8 IU/L) (reference range, 1.5-12.4 mIU/mL [SI: 1.5-12.4 IU/L]), luteinizing hormone <0.07 mIU/mL (SI: <0.07 IU/L) (reference range, 1.7-8.6 mIU/mL [SI: 1.7-8.6 IU/L]), and estradiol <12 pg/mL (SI: <44 pmol/L) (reference range, 5-36 pg/mL [SI: 18-132 pmol/L]) (Table 1). Given the patient's age and absent pubertal development, these results were described as prepubertal rather than as definitive hypogonadotropic hypogonadism. However, during subsequent follow-up at 13 years of age, the patient exhibited a clear delay in the development of secondary sexual characteristics alongside persistently suppressed gonadotropins. At this point, the diagnosis of hypogonadotropic hypogonadism was established, prompting the initiation of estrogen replacement therapy (later transitioned to cyclic estrogen-progestogen therapy) to induce puberty and support bone health.

The plasma GHRH concentration was markedly elevated at 962 ng/dL (SI: 9620 ng/L) (reference range, <60 ng/dL [SI: <600 ng/L]) (Table 1), supporting ectopic GHRH secretion. Twenty-four-hour urinary 5-hydroxyindoleacetic acid excretion was normal at 9.2 mg/24 hour (SI: 48 µmol/24 hour) (reference range, <12 mg/24 hour [SI: <63 µmol/24 hour]) (Table 1). Brain magnetic resonance imaging (MRI) showed diffuse pituitary enlargement with suprasellar extension and optic chiasm displacement, consistent with pituitary hyperplasia secondary to chronic GHRH stimulation rather than a discrete adenoma (Fig. 1C).

Although multiplex ligation-dependent probe amplification (MLPA) analysis did not detect copy-number alterations involving the MEN1 gene, a clinical diagnosis of MEN1 remains possible. Throughout the 23-year follow-up, the patient has lacked any additional clinical or biochemical features indicative of the syndrome. Serial laboratory evaluations have consistently demonstrated normal serum calcium and phosphorus levels, effectively ruling out primary hyperparathyroidism. Furthermore, repeated abdominal imaging has shown a morphologically normal pancreas with no evidence of pancreatic neuroendocrine tumors, and the patient has never exhibited symptoms of Zollinger–Ellison syndrome. Dermatological examinations have revealed no cutaneous angiofibromas or collagenomas, and there is no family history of MEN1-associated endocrine tumors.

## Treatment

At 12 years of age, because of tumor progression, a complete surgical resection of the primary tumor bed was performed, and pathology confirmed metastatic lymphadenopathy from neuroendocrine tumor. Interferon-αwas attempted without response.

Treatment with first-generation somatostatin analogs (SSAs) and dopamine agonist therapy was then initiated to control GH/IGF-1 and prolactin hypersecretion. The regimen included octreotide long-acting release (LAR) at a dose of 30 mg every 28 days and bromocriptine, which was later switched to cabergoline 0.5 mg weekly. Estrogen-progestogen replacement therapy was added to induce puberty and support bone health.

Because metastatic disease persisted with morphologic and biochemical progression and high somatostatin receptor expression (Krenning score 3 on Octreoscan and gallium-68 DOTA-peptide positron emission tomography/computed tomography [PET/CT]) (Fig. 1D), peptide receptor radionuclide therapy (PRRT) with lutetium-177 DOTA-Tyr3-octreotate (^177^Lu-DOTATATE) was administered. Seven initial cycles, with a total cumulative dose of 682 mCi, were given between 16 and 18 years of age at the European Institute of Oncology in Milan, achieving radiologic stability.

Despite radiologic stability during high-dose octreotide therapy and the addition of pegvisomant, which was initiated in 2012 at 19 years of age and discontinued in November 2013 at 20 years of age, biochemical control of GH excess remained inadequate. At 20 years of age, treatment was switched to pasireotide LAR 60 mg intramuscularly every 28 days as off-label therapy. This change was associated with stabilization of IGF-1 and GH concentrations and improved clinical control of hypersomatotropism.

## Outcome and follow-up

At 22 years of age, the patient developed severe dorsal kyphoscoliosis, likely exacerbated by rapid linear growth associated with uncontrolled gigantism, with a restrictive pattern on pulmonary function testing. She required extensive surgical correction by posterior spinal arthrodesis and instrumentation.

At 26 years of age, because of persistent metabolically active tumor burden, 2 additional ^177^Lu-DOTATATE doses of 200 mCi each were administered. Serial gallium-68 DOTA0-Phe1-Tyr3-octreotide (68Ga-DOTATOC) PET/CT scans showed stable disease through 31 and 32 years of age.

At the most recent evaluation, at 32 years of age, the patient remained in good general condition, with Eastern Cooperative Oncology Group performance status 0 and Karnofsky Performance Status 100%. Her regimen included pasireotide 60 mg monthly, cabergoline, statin therapy for familial hypercholesterolemia, and metformin. The serum chromogranin A concentration was 243.6 ng/mL (SI: 243.6 µg/L) (reference range, 93-311 ng/mL [SI: 12.2-40.7 nmol/L]), and the serum IGF-1 concentration was 163 ng/mL (SI: 21.3 nmol/L) (reference range, <100 ng/dL [SI: <600 µg/L]) (Table 1). The somatotropic axis and tumor burden have remained stable since initiation of pasireotide.

## Discussion

Ectopic GHRH secretion is a rare cause of hypersomatotropism, accounting for less than 1% of acromegaly and gigantism cases [3]. Bronchial carcinoids and pancreatic neuroendocrine tumors are the most frequently reported sources of ectopic GHRH in adults [6]. Pediatric-onset cases are exceptional, and previously described pediatric cases have mainly involved pancreatic neuroendocrine tumors associated with MEN1 [5]. The growth chart (Fig. 2) documents height tracking at and above the 97th percentile, with a final stature markedly exceeding the mid-parental target, supporting GHRH-driven growth acceleration as the dominant driver of the gigantism.

This case adds to the limited literature on childhood-onset ectopic GHRH secretion by showing that a bronchial neuroendocrine tumor can cause severe GH excess and metastatic disease in childhood. The case is notable for long-term survival and disease stability over more than 20 years despite stage IV bronchial neuroendocrine tumor, although claims regarding MEN1 exclusion should be interpreted in light of the need to document the clinical MEN1 phenotype, not only the negative genetic result.

Diagnostically, the presence of diffuse pituitary hyperplasia without a discrete adenoma on MRI is suggestive of GHRH stimulation. Measurement of plasma GHRH should be considered in this setting because GHRH can be secreted by a neuroendocrine malignancy. This distinction is clinically important because pituitary hyperplasia can be misidentified as a macroadenoma, leading to unnecessary pituitary surgery that does not address the ectopic source and may cause hypopituitarism [6, 7]. Screening for ectopic GHRH secretion should therefore be considered before neurosurgical intervention when the imaging pattern and clinical context are compatible with GHRH-driven disease.

Therapeutic management required control of both hormonal excess and metastatic tumor burden. First-generation SSAs, including octreotide and lanreotide, primarily target somatostatin receptor subtype 2 (SSTR2) [8]. In this patient, octreotide and pegvisomant were associated with radiologic stability but inadequate biochemical control of GH excess, rather than true pharmacologic resistance. Similar challenges have been described in ectopic GHRH cases, in which hormonal control may be influenced by tumor burden and somatostatin receptor expression [4, 9, 10].

Pasireotide, a multiligand SSA with high affinity for somatostatin receptor subtype 5 in addition to SSTR2, proved useful for biochemical control in this patient. This observation is consistent with the rationale for pasireotide in selected cases of GH excess when first-generation SSAs do not adequately control IGF-1 or GH concentrations [11].

Peptide receptor radionuclide therapy with ^177^Lu-DOTATATE also contributed to long-term structural disease control. Although PRRT was initially established most strongly in gastroenteropancreatic neuroendocrine tumors, evidence supporting its use in somatostatin receptor–positive bronchial neuroendocrine tumors has increased [12, 13]. In this patient, PRRT was incorporated into a multimodal sequence that included surgery and medical therapy and was followed by prolonged structural stability.

The severe kyphoscoliosis in this case underscores the effect of uncontrolled GH/IGF-1 excess on the developing skeleton [2, 14]. Early recognition and aggressive multidisciplinary management are essential to reduce irreversible skeletal, metabolic, and functional complications in pediatric gigantism.

This report has limitations inherent to a long-term retrospective case description. Mid-parental target height percentile, the original growth chart, detailed clinical MEN1 screening data, and exact pegvisomant dates require author confirmation or should be explicitly reported as unavailable. Detailed growth data before 7 years of age were not available owing to the long retrospective follow-up; nonetheless, the available later-childhood records (Fig. 2) document sustained growth above the 97th percentile. Nevertheless, the case illustrates the diagnostic value of recognizing GHRH-driven pituitary hyperplasia and the potential for prolonged stabilization with coordinated endocrine, oncologic, nuclear medicine, and surgical care.

In summary, ectopic GHRH secretion should be considered in pediatric patients with gigantism or acromegalic features when pituitary MRI demonstrates diffuse hyperplasia without a discrete adenoma. Plasma GHRH measurement can prevent unnecessary pituitary surgery and redirect treatment toward the neuroendocrine source. Long-term disease control may be achievable with multimodal treatment, including SSAs, pasireotide in selected patients, and PRRT for somatostatin receptor–positive metastatic disease.

## Learning points

- In pediatric gigantism or acromegaly with diffuse pituitary hyperplasia and no discrete pituitary adenoma, ectopic GHRH secretion should be considered, and plasma GHRH measurement should be obtained before pituitary surgery when clinically appropriate.
- A negative MEN1 genetic test does not by itself exclude MEN1; clinical assessment should document calcium/parathyroid hormone status, pancreatic neuroendocrine tumor or Zollinger–Ellison syndrome evaluation, cutaneous manifestations, and family history.
- Pasireotide may improve biochemical control of ectopic GH excess when first-generation SSAs and pegvisomant provide inadequate control, but biochemical response should be distinguished from radiologic tumor stability.
- Peptide receptor radionuclide therapy with ^177^Lu-DOTATATE can contribute to long-term structural disease control in metastatic bronchial neuroendocrine tumors with high somatostatin receptor expression.

## Contributors

Drafting of the manuscript and conceptualization: F.V.-O.D.L. and M.I.D.O.-G. Critical revision and supervision of the manuscript: D.S.-P., L.H.-R., and M.I.D.O.-G. All authors contributed to clinical management. All authors reviewed and approved the final version of the manuscript for publication.

## Data Availability

No datasets were generated or analyzed for this report. Additional clinical details are not publicly available because they could compromise patient privacy.

## References

[1] Giustina A, Colao A. Acromegaly. N Engl J Med. 2025;393(19):1926‐1939. 10.1056/NEJMra2409076 41223366

[2] Wydra A, Stelmachowska-Banaś M, Czajka-Oraniec I. Skeletal complications in acromegaly. Rheumatology. 2023;61(4):248‐255. 10.5114/reum/169918 PMC10515128 37745143

[3] Zendran I, Gut G, Kałużny M, Zawadzka K, Bolanowski M. Acromegaly caused by ectopic growth hormone-releasing hormone secretion: a review. Front Endocrinol (Lausanne). 2022;13:867965. 10.3389/fendo.2022.867965 PMC9218487 35757397

[4] Lamback EB, Henriques DG, Vazquez-Borrego MC, et al Growth hormone-releasing hormone-secreting pulmonary neuroendocrine tumor associated with pituitary hyperplasia and somatotropinoma. Arch Endocrinol Metab. 2021;65:648‐663. 10.20945/2359-3997000000395 PMC10528565 34591404

[5] Srirangam Nadhamuni V, Iacovazzo D, Evanson J, et al GHRH secretion from a pancreatic neuroendocrine tumor causing gigantism in a patient with MEN1. Endocrinol Diabetes Metab Case Rep. 2021;2021:EDM200208. 10.1530/EDM-20-0208 PMC8240703 34156350

[6] Fainstein-Day P, Ullmann TE, Dalurzo MCL, Sevlever GE, Smith DE. The clinical and biochemical spectrum of ectopic acromegaly. Best Pract Res Clin Endocrinol Metab. 2024;38(3):101877. 10.1016/j.beem.2024.101877 38413286

[7] Ghazi AA, Amirbaigloo A, Dezfooli AA, et al Ectopic acromegaly due to growth hormone-releasing hormone. Endocrine. 2013;43(2):293‐302. 10.1007/s12020-012-9790-0 PMC3553305 22983831

[8] Gomes-Porras M, Cárdenas-Salas J, Álvarez-Escolá C. Somatostatin analogs in clinical practice: a review. Int J Mol Sci. 2020;21(5):1682. 10.3390/ijms21051682 PMC7084228 32121432

[9] Gudbjartsson T, Agnarsson BA, Palsson PS, Johannesson A. Acromegaly caused by ectopic growth hormone-releasing hormone production from a bronchial carcinoid tumor. Thorac Cardiovasc Surg. 2011;59(3):184‐185. 10.1055/s-0030-1250341 21480145

[10] Besser GM, Burman P, Daly AF. Predictors and rates of treatment-resistant tumor growth in acromegaly. Eur J Endocrinol. 2005;153(2):187‐193. 10.1530/eje.1.01968 16061822

[11] Cuevas-Ramos D, Fleseriu M. Pasireotide: a novel treatment for patients with acromegaly. Drug Des Devel Ther. 2016;10:227‐239. 10.2147/DDDT.S77999 PMC4714742 26811671

[12] Brabander T, van der Zwan WA, Teunissen JJM, et al Long-term efficacy, survival, and safety of [177Lu-DOTA0,Tyr3]octreotate in patients with gastroenteropancreatic and bronchial neuroendocrine tumors. Clin Cancer Res. 2017;23(16):4617‐4624. 10.1158/1078-0432.CCR-16-2743 28428192

[13] Naik M, Al-Nahhas A, Khan SR. Treatment of neuroendocrine neoplasms with radiolabeled peptides: where are we now. Cancers (Basel). 2022;14(3):761. 10.3390/cancers14030761 PMC8833798 35159027

[14] Gurnurkar S, Patel U, Seekford J, Carakushansky M, Chegondi M. Two unusual cases of pituitary tumors presenting with pediatric acromegaly. Cureus. 2022;14:e31604. 10.7759/cureus.31604 PMC9757648 36540490

